# What shapes 7-year-olds’ subjective well-being? Prospective analysis of early childhood and parenting using the Growing Up in Scotland study

**DOI:** 10.1007/s00127-016-1246-z

**Published:** 2016-06-30

**Authors:** Alison Parkes, Helen Sweeting, Daniel Wight

**Affiliations:** MRC/CSO Social and Public Health Sciences Unit, University of Glasgow, Top Floor, 200, Renfield Street, Glasgow, G2 3QB UK

**Keywords:** Child subjective well-being, Parenting, Life satisfaction, Poverty, Mental health, Rural health

## Abstract

**Purpose:**

Research on predictors of young children’s psychosocial well-being currently relies on adult-reported outcomes. This study investigated whether early family circumstances and parenting predict 7-year-olds’ subjective well-being.

**Methods:**

Information on supportive friendships, liking school and life satisfaction was obtained from 7-year-olds in one Growing Up in Scotland birth cohort in 2012–2013 (*N* = 2869). Mothers provided information on early childhood factors from 10 to 34 months, parenting (dysfunctional parenting, home learning and protectiveness) from 46 to 70 months, and 7-year-olds’ adjustment. Multivariable path models explored associations between early childhood factors, parenting and 7-year-olds’ subjective well-being. Supplementary analyses compared findings with those for mother-reported adjustment.

**Results:**

In a model of early childhood factors, maternal distress predicted less supportive friendships and lower life satisfaction (coefficients −0.12), poverty predicted less supportive friendships (−0.09) and remote location predicted all outcomes (−0.20 to −0.27). In a model with parenting added, dysfunctional parenting predicted all outcomes (−10 to −0.16), home learning predicted liking school (0.11) and life satisfaction (0.08), and protectiveness predicted life satisfaction (0.08). Effects of maternal distress were fully mediated, largely via dysfunctional parenting, while home learning mediated negative effects of low maternal education. Direct effects of poverty and remote location remained. Findings for mother-reported child adjustment were broadly similar.

**Conclusions:**

Unique prospective data show parenting and early childhood impact 7-year-olds’ subjective well-being. They underline the benefits for children of targeting parental mental health and dysfunctional parenting, and helping parents develop skills to support children at home and school.

**Electronic supplementary material:**

The online version of this article (doi:10.1007/s00127-016-1246-z) contains supplementary material, which is available to authorized users.

## Introduction

Over the last decade, there has been growing recognition of the importance of understanding children’s subjective well-being. Subjective well-being covers both affective and cognitive dimensions, respectively concerned with experiencing emotions and evaluating one’s life, including overall life satisfaction [1]. Young children’s emotional states may be relatively transitory, so in common with most research we focus on cognitive aspects of subjective well-being here. Children’s own perspectives permit corroboration of adult-reported socio-emotional development, and are likely to provide unique insights on needs and priorities that could otherwise be overlooked. Secondary school-age children’s views are now routinely included in cross-national comparisons of overall child well-being, and although rankings of life satisfaction are broadly associated with objective socio-economic indicators [2], there are poorly understood anomalies. For instance, subjective life satisfaction is generally associated with public expenditure on families and education, but in the Netherlands, children appear much happier than might be predicted from their country’s spending [3]. This has led to debate on whether social policies can really improve children’s happiness. Early intervention may be most cost-effective, but as a first step we need to be able to ascertain the main determinants of subjective well-being from early childhood. Our current knowledge of subjective well-being is mainly based on children aged 10 or older and lacks prospective information from the early years (though see two recent cross-sectional studies of 7- and 9-year-olds [4, 5]). Data collected by Growing Up in Scotland (GUS), a large birth cohort study enables us, for the first time, to investigate the influence of early family circumstances on the views of young children themselves. To explore possible origins of social inequalities in young children’s subjective well-being, our study develops an ecological risk model [6].

### Family risk factors

Theoretical and empirical work suggests that social relationships, health and income constitute important determinants of life satisfaction among adults, with evidence from a number of countries pointing to the primacy of social relationships [7]. Our ecological model focuses on the family setting, being generally where the first foundations will be laid for social relationships critical to future happiness; indeed, happiness with family life was the most important correlate of life satisfaction in a large UK study of 8- to 17-year-olds [8]. We draw on key parental and household resources already established as important for children’s mental health. Several indicators of low family economic and psychological resources, such as poverty, poor parental health, substance use, low parental education and single parenthood, are often found to co-occur, but may nonetheless represent independent threats to socio-emotional adjustment [9–12]. There is some evidence that parental mental health [13, 14], substance use [15] and socio-economic status [16], together with family income [17–19] and structure [20–22], also affect older children’s subjective well-being. However, almost all the studies on older children have had a cross-sectional design; and findings in relation to factors found important across studies are inconsistent. Further longitudinal studies, including on younger age groups, are required to supplement this evidence base and inform our understanding of how social inequalities in children’s subjective well-being may develop.

### Home location risk factors

We extend the ecological model beyond parent and household characteristics to consider whether home location influences young children’s subjective well-being. As for family setting, we select aspects of location already implicated in young children’s socio-emotional adjustment. Area deprivation has been linked to poor socio-emotional adjustment in several studies of young children [23, 24] although in some instances its effects are explained by family socio-economic status [25]. Less is known about the effects of urban–rural location on young children’s adjustment. Some research has shown differences in behavioural and emotional problems according to degree of urbanisation. One US study found more behavioural problems in rural areas [26], while an English study found fewer behavioural and peer problems in less sparse rural areas compared to either more urban or predominantly rural areas, and fewer emotional problems in areas with an urban/rural mix compared to other area types [27]. Elsewhere, clear urban–rural differences have not been found [28, 29]. In relation to area effects on subjective well-being, again we find that existing studies are confined to older age groups. An ecological assets model of adolescent life satisfaction has underlined the importance of social relationships in different domains including school and neighbourhood as well as the family [30], while limited research on the effects of urbanisation suggests lower adolescent subjective well-being among rural populations [31, 32].

### Pathways from family and home location risk factors via parenting

Our ecological model is developed further by examining possible pathways from early childhood influences to children’s subjective well-being, via parenting. Family processes, through which a child develops and sustains relationships with parents, and others, are theorised as dependent on contextual stress and support [33]. In selecting family processes as potential mediators of early childhood factors within the family setting, we draw on two models seeking to explain socio-economic inequalities in children’s development: family investment and family stress [34]. In the case of family investment, economic resources are posited as directly affecting the quality of parenting, extending beyond financial investment to cover parental involvement with the child’s upbringing, including time spent with the child. In the family stress model, economic resources are posited as affecting the quality of parenting indirectly, via parental mental health. In relation to the family investment model, we examine home learning, such as looking at books, drawing and singing. These activities help children adjust to school learning [35], and are likely to bolster emotional engagement with school. Importantly, however, research has also suggested a connection between parental provisions of enjoyable stimulation and reduced socio-emotional problems, as well as mediation of the effects of early socio-economic disadvantage on these outcomes [36–39]. This suggests that parental investment in home learning may help promote increased subjective well-being more generally (i.e. beyond any effect on school adjustment), perhaps because children are less bored and frustrated, and have a greater sense of being nurtured. Second, in relation to the family stress model, we examine dysfunctional parenting characterised by multiple challenges, such as high levels of parent–child conflict, parenting stress and a chaotic home environment. These aspects of dysfunctional parenting have been found to be associated with child behavioural and emotional problems, as well as channelling some of the associations found for socio-economic disadvantage, single parenthood or poor parental mental health with these problems [11, 40–43]. It is therefore plausible to suggest that dysfunctional parenting may lead to lower child subjective well-being, as well as providing a pathway linking early childhood disadvantage with well-being.

Looking beyond the family setting, neighbourhood characteristics may also shape parenting in various ways. Local institutions and resources may facilitate better parenting via provision of health and welfare services, stimulating environments, such as libraries, parks and sports facilities, and opportunities for socialisation. In addition, other adults living in the neighbourhood may provide families with role models and emotional or instrumental support for parenting. Evidence that neighbourhood effects on children’s well-being may operate via parenting comes from a US study, where effects of neighbourhood deprivation on children’s socio-emotional adjustment, independent of family-level socio-economic status and risk, were mediated by less supportive parenting [44]. In our study, we might expect some area-level influences on children’s subjective well-being to be explained via processes linked to family investment and stress. However, neighbourhood deprivation and urbanisation may also be associated with differences in parental perceived safety in relation to social disorder, “stranger danger” and road traffic, leading to differences in children’s outdoor physical activity [45, 46]. We therefore consider a third aspect of parenting, protectiveness of the child playing outdoors, which may provide an additional pathway for area effects on children’s subjective well-being linked to parental safety concerns. Parental protectiveness might itself exert some negative influence on well-being, by limiting children’s independence and their ability to form friendships with children outside school hours. Alternatively, and especially for young children, parental protectiveness is likely to enhance perceptions of being valued and cared for, and may shield them from adverse or challenging situations: consequently, protectiveness seems more likely to be associated with greater, rather than lower, overall child subjective well-being.

### Alternative pathways from family and home location risk factors

Although parenting processes may channel many of the effects of early childhood on school-age children’s subjective well-being, it is likely that stable family circumstances will also over time begin to affect the child in other ways. Alternative mechanisms for effects of family-level factors such as living in poverty or without a resident father could involve social comparison, although young children may not have highly developed expectations, and may be relatively unaware of differences between their own family circumstances and those of other children [47]. It has also been argued that children’s natural resilience may allow adaptation to all, but the most extreme family circumstances [48]. In the case of poverty, qualitative research with low-income families suggests children of all ages acknowledge its impact, but they also show adaptation to its effects [49].

There may also be other mechanisms for effects of home location on subjective well-being. Some positive pathways from lower levels of neighbourhood deprivation or urbanisation have been found. Both lower area deprivation and moderate rurality in England have been found to influence children’s socio-emotional adjustment, via improved school quality [24, 27]. A Swedish study found children’s lower perceptions of community trust and safety in urban areas was associated with lower subjective well-being, compared to rural areas [50]. Rurality might also benefit children via closer proximity to green spaces; but although there is some evidence of benefits of green spaces within residential environments for adult mental health, little is known in relation to children [51]. Moreover, a recent review has challenged adult perspectives of the idyllic rural childhood, finding that children often see the rural environment as restrictive and unsafe [52]. Negative pathways might also exist from remoteness to children’s subjective well-being. Pathways linked to increased travel times might involve social isolation and poor access to health services implicated in adult studies [53, 54]. Social comparisons may also have a role to play if children in remote areas are sensitive to differences in their lives compared to the vast majority living near urban centres.

Our study investigates early childhood and parenting predictors of three aspects of 7-year-olds’ subjective well-being: overall life satisfaction, enjoyment of school and supportive friendships. Based on the existing research on children’s psychosocial adjustment, we hypothesise that the two strongest early predictors of lower subjective well-being will be maternal distress (poor mental health and substance use), and family poverty. As the ecological model of child development puts parents at the core of young children’s socialisation, we expect that more proximal effects of parenting processes will, to a large extent, channel the more distal effects of early life factors on subjective well-being. Our focus is on children’s own views, but since these are relatively untested for this young age group we also explore mothers’ reports of child adjustment in supplementary analyses.

## Methods

Data were from the first birth cohort of the Growing Up in Scotland study, a nationally representative cohort of families with children born between June 2004 and May 2005. Details of the sampling framework are provided elsewhere [55]. Baseline data were gathered from 5217 families during 2005–2006, when children were 10 months old, and these families were followed up annually for 5 years (to 70 months), and then after 2 years (94 months, *N* = 3,456). This study used information obtained in 2012–2013 from computer-assisted personal interviews conducted with the main carer from 10 to 70 months; and from the cohort child at 94 months using an audio computer-assisted self-completion questionnaire. The analysis sample was restricted to cases where the child completed a questionnaire at 94 months, the child was a singleton birth, and the mother was the main carer interviewed at all previous survey sweeps (*N* = 2869).

### Measures

#### Outcome measures

Child-reported information at 94 months was used to create three latent (not directly observed) constructs related to subjective well-being. Life satisfaction used five items from Huebner’s Student Life Satisfaction Scale [56] concerning whether the child feels he/she has a good life, has what he/she wants in life, his/her life is just right, wishes life was different, and feels life is going well (indicator loadings 0.46–0.68). Liking school and supportive friendships respectively used items from the school and friends domains of the Multidimensional Life Satisfaction Scale [57]; liking school used three items (“I enjoy learning at school”, “I hate school”, “I look forward to going to school”, loadings 0.64–0.84); supportive friendships used two items (“My friends are mean to me”, “My friends are nice to me”, loadings 0.71 and 0.48).

Mother-reported outcomes at the 94 month interview used the five-item subscales of the Strengths and Difficulties Questionnaire [58] to measure child emotional problems and child peer relationship problems, Cronbach’s alphas 0.68 and 0.65, respectively; and two items designed specifically for the study to measure school adjustment, based on how often the child looked forward to going to school, and how often he/she was reluctant to go (alpha = 0.50).

#### Main predictors

These are outlined below. Further details are provided in a supplementary file (S1), which also provides information on additional covariates included in the analysis as potential confounders of predictor-outcome associations (child gender, first born status, general health at 10–22 months, developmental delay at 22 months, and cognitive score at 34 months; maternal ethnic group, age at birth of the child and low physical health at 10 months; and the number of children in the household at 10 months).

Maternal education was reported by mothers at 10 months and classified into four levels: the lowest group comprised mothers obtaining lower-level qualifications at school leaving age, or no qualifications at all; and the highest group comprised mothers with degree level qualifications. Maternal distress was modelled as a latent construct using indicators of poor maternal mental health (10, 22, and 34 months) and illegal drug use (10 and 34 months). Consideration of alternative measurement models indicated that the optimal fit was achieved by a single construct for mental health and drug use. Other available indicators covering smoking and alcohol use were explored, but rejected due to insufficiently high factor loadings, i.e. <0.4. Father absence was defined as the father being absent from the household at one or more of the 10, 22 and 34 month surveys. Family poverty was a latent construct using indicators of low family income (≤60 % of UK median income) and workless household at the 10, 22, and 34 month surveys. Area deprivation was measured by linking to the Scottish Index of Multiple Deprivation via home postcode. Two alternative groupings of the Scottish Urban/Rural Classification also linked to home postcode at the 10 month survey were explored: rurality and remoteness. Both groupings contained large urban areas as a reference, and had other urban areas as one comparison group. To explore rurality, we examined two further comparison groups: small towns (combined accessible/remote) and rural (combined accessible/remote). To explore remoteness, these groups were replaced with two alternative groupings: accessible (combined small town/rural) and remote (combined small town/rural). For more details, see Supplementary File S1.

For parenting, home learning was a latent construct based on four home activities at 46, 58, and 70 months: how often the child looked at books, drew pictures, sang nursery rhymes and played at recognising letters and numbers. Dysfunctional parenting was a latent construct based on parent–child conflict, home disorganisation and parenting stress (all at 58 months). Protectiveness was measured directly, based on four items relating to looking after the child playing outdoors (46 months).

### Statistical analysis

Structural equation modelling used Mplus version 7.3 [59]. Missing item response was generally extremely low (<1 %) with the exception of information on income, developmental delay and cognitive score (4–6 %). To reduce bias and increase statistical power, missing information was imputed using Mplus multiple imputation facility. Analyses used weighted least squares means and variance adjusted (WLSMV) estimation, combining results across 20 imputed data sets. They allowed for the complex survey design and used survey weights to counteract the effects of differential attrition from baseline to 94 months (35 % overall, but 53 % among mothers with low education, 55 % among families with no resident father and 52 % among families reporting the lowest quintile of household income at baseline). There were two main stages to the analyses. First, multivariable regression models explored associations between early childhood factors and 7-year-olds’ outcome measures, including terms to specify covariance between early childhood factors. Second, path models explored whether parenting mediated effects of early childhood factors on child outcome measures. The Mplus Model Indirect function was used to estimate indirect effects of early childhood factors on child outcomes via parenting. Throughout, statistical significance was set at *p* < 0.05.

## Results

The distribution of 7-year-olds’ views with respect to questionnaire items on liking school, supportive friendships and overall life satisfaction are shown in Table 1. Most gave positive views of their lives: over all ten items, the proportion supplying one of the two less favourable response options ranged from 7 % “often” or “always” thinking “my friends are mean to me” to 44 % “sometimes” or “never” reporting “I look forward to going to school”. The three latent constructs were significantly correlated with one another. Supportive friends and liking school were more strongly correlated with overall life satisfaction (0.46 and 0.44, respectively) than with each other (0.27).

**Table 1:** Seven-year-olds’ views on life satisfaction, supportive friendships and liking school: distribution of responses

Latent construct	Indicator items	Distribution of responses (row %)			
		Never	Sometimes	Often	Always
Life satisfaction	Do you feel that your life is going well?	3.4	13.5	20.9	62.2
	Do you wish your life was different?	68.1	21.8	4.5	5.6
	Do you feel that your life is just right?	4.5	17.5	19.6	58.5
	Do you feel you have what you want in life?	8.2	25.3	23.5	42.9
	Do you feel you have a good life?	3.6	11.7	14.9	69.8
Supportive friendships	My friends are nice to me	1.3	12.4	17.0	69.4
	My friends are mean to me	61.6	31.7	3.1	3.6
Liking school	I look forward to going to school	16.5	27.3	17.6	38.6
	I hate school	58.6	23.8	6.1	11.6
	I enjoy learning at school	10.1	18.9	17.0	54.0

Unadjusted associations for early childhood factors, parenting and covariates with child- reported outcomes are shown in Table 2. Low maternal education was associated with less liking of school. Maternal distress and family poverty were associated with the child reporting less supportive friendships and lower life satisfaction, with an additional borderline (*p* = 0.07) association between maternal distress and disliking school. Father absence, area deprivation and rural location were not clearly associated with child outcomes, but remote location was negatively associated with child-reported life satisfaction and liking school (and borderline, *p* = 0.09 for supportive friendships). Home learning was positively associated with life satisfaction and liking school, protectiveness was positively associated with life satisfaction, and dysfunctional parenting was negatively associated with all three outcomes.

**Table 2:** Early childhood, parenting and covariate measures: unadjusted associations with 7-year-olds’ subjective well-being

Measure, timing and reference group (for categorical measures)	contrast/effect	Child-reported outcomes at 94 months					
		Supportive friendships		Liking school		Life satisfaction	
		*β *(SE)	*p*	*β* (SE)	*p*	*β* (SE)	*p*
Early childhood (10–34 months)							
Maternal factors							
Educational level 10 months (degree)	Advanced	0.00 (0.05)	0.928	−0.02 (0.06)	0.733	0.01 (0.05)	0.768
	Intermediate	−0.08 (0.06)	0.209	0.03 (0.06)	0.681	−0.07 (0.07)	0.310
	low	−0.05 (0.10)	0.597	**−0.20 (0.09)**	**0.019**	−0.08 (0.10)	0.408
Distress 10–34 months	Higher	**−0.16 (0.04)**	**<0.001**	−0.07 (0.04)	0.074	**−0.16 (0.04)**	**<0.001**
Household factors							
Family poverty 10–34 months	Higher	**−0.09 (0.04)**	**0.014**	−0.04 (0.03)	0.283	**−0.12 (0.04)**	**0.001**
Father absence 10–34 months (no)	Yes	0.03 (0.08)	0.743	−0.07 (0.07)	0.329	−0.10 (0.08)	0.221
Geographical factors							
Area deprivation	Higher	−0.01 (0.02)	0.694	−0.01 (0.02)	0.405	−0.03 (0.02)	0.119
Rurality^1^ 10 months (large urban)	other urban	0.06 (0.07)	0.387	0.06 (0.06)	0.263	0.02 (0.07)	0.816
	Small town	0.00 (0.09)	0.971	0.01 (0.07)	0.843	0.03 (0.08)	0.726
	Rural	−0.11 (0.07)	0.128	−0.10 (0.05)	0.051	−0.11 (0.06)	0.065
Remoteness^1^ 10 months (large urban)	Other urban	0.06 (0.07)	0.387	0.06 (0.06)	0.263	0.02 (0.07)	0.816
	Accessible	−0.04 (0.07)	0.589	0.00 (0.05)	0.958	0.00 (0.07)	0.947
	Remote	−0.14 (0.08)	0.085	**−0.22 (0.07)**	**0.002**	**−0.21 (0.06)**	**<0.001**
Parenting (46–70 months)							
Home learning 46–70 months	More frequent	0.04 (0.03)	0.289	**0.22 (0.03)**	**<0.001**	**0.15 (0.03)**	**<0.001**
Dysfunctional parenting 58 months	Higher	**−0.16 (0.04)**	**<0.001**	**−0.13 (0.03)**	**<0.001**	**−0.23 (0.04)**	**<0.001**
Protectiveness 46 months	Higher	−0.03 (0.04)	0.432	0.03 (0.03)	0.318	**0.10 (0.03)**	**0.001**
Covariates							
Child factors							
Gender (male)	Female	**0.18 (0.05)**	**<0.001**	**0.48 (0.05)**	**<0.001**	**0.20 (0.05)**	**<0.001**
First born (no)	Yes	−0.02 (0.06)	0.700	**0.15 (0.05)**	**0.005**	0.10 (0.06)	0.066
General health 10–22 months	Worse	−0.02 (0.06)	0.803	−0.02 (0.06)	0.701	−0.09 (0.05)	0.085
Developmental delay 22 months (no)	Yes	**−0.35 (0.14)**	**0.010**	−0.16 (0.12)	0.173	**−0.33 (0.12)**	**0.008**
Cognitive score 34 months	higher	**0.01 (0.00)**	**0.011**	**0.01 (0.00)**	**0.023**	**0.01 (0.00)**	**0.008**
Maternal factors							
Ethnic group (white)	Minority	−0.18 (0.15)	0.204	0.09 (0.12)	0.443	−0.17 (0.11)	0.116
Age at birth of child (30–39 years)	<20 years	−0.12 (0.15)	0.432	0.07 (0.13)	0.586	0.05 (0.12)	0.686
	20–29 years	0.01 (0.06)	0.911	0.04 (0.05)	0.431	−0.08 (0.06)	0.145
	40 years or older	0.03 (0.12)	0.818	0.07 (0.12)	0.547	−0.09 (0.09)	0.354
Low physical health 10 months (no)	Yes	**−0.22 (0.10)**	**0.031**	−0.05 (0.08)	0.579	−0.14 (0.11)	0.198
Household factors							
Number of children in household 10 months (one)	Two	0.01 (0.07)	0.902	−0.13 (0.06)	0.018	−0.07 (0.07)	0.266
	Three	0.05 (0.08)	0.549	**−0.25 (0.08)**	**0.001**	−0.12 (0.09)	0.172
	Four or more	0.13 (0.14)	0.372	**−0.27 (0.13)**	**0.034**	−0.17 (0.12)	0.183

Unstandardised coefficients and standard errors (SE) are shown, with probability *p.* Figures in bold show associations that were statistically significant at the *p* < 0.05 level.^1^Two alternative groupings of the Scottish urban–rural indicator are shown for non-urban areas. The first divides non-urban locations into small towns and rural, and the second divides them into accessible and remote (for further details, see Supplementary File S1)

Table 3 shows results of multivariable models. Stage 1 models associations between early childhood factors and child-reported outcomes, adjusting for child gender, birth order, health, developmental delay and cognitive score; mother’s ethnicity, age at birth of child and low physical health; and number of children in the household (covariates not shown). In these models, covariance between family poverty, area deprivation and maternal distress ranged from 0.25 to 0.52 (not shown in Table, all *p* < 0.001). Maternal distress and family poverty were negatively associated with supportive friendships and lower life satisfaction (borderline for the effect of poverty on life satisfaction, *p* = 0.075). Remote location was negatively associated with all three outcomes.

**Table 3:** Early childhood and parenting measures: adjusted associations with seven-year-olds’ subjective well-being

Measure (with reference group for categorical measures)	Effect/contrast	Child-reported outcomes at 94 months											
		Supportive friendships				Liking school				Life satisfaction			
		Stage 1		Stage 2		Stage 1		Stage 2		Stage 1		Stage 2	
		*β* (SE)	*p*	*β* (SE)	*p*	*β* (SE)	*p*	*β* (SE)	*p*	*β *(SE)	*p*	*β* (SE)	*p*
Early childhood (10–34 months)													
Maternal education (degree)	Advanced	−0.02 (0.06)	0.774	−0.04 (0.06)	0.493	0.00 (0.06)	0.957	−0.02 (0.06)	0.775	0.07 (0.06)	0.227	0.04 (0.06)	0.472
	Intermediate	−0.10 (0.07)	0.138	−0.11 (0.07)	0.105	0.04 (0.07)	0.556	0.07 (0.07)	0.350	0.02 (0.08)	0.777	0.03 (0.08)	0.659
	Low	−0.08 (0.10)	0.386	−0.07 (0.10)	0.508	−0.13 (0.09)	0.123	−0.06 (0.09)	0.536	0.07 (0.09)	0.426	0.15 (0.10)	0.113
Maternal distress (lower)	Higher	−**0.12 (0.04)**	**0.001**	−0.07 (0.05)	0.223	−0.04 (0.04)	0.223	0.03 (0.05)	0.480	−**0.12 (0.04)**	**0.002**	−0.01 (0.05)	0.891
Family poverty (lower)	Higher	−**0.09 (0.04)**	**0.013**	−**0.08 (0.04)**	**0.022**	0.02 (0.03)	0.492	0.03 (0.04)	0.385	−0.07 (0.04)	0.075	−0.06 (0.04)	0.137
Absent father (no)	Yes	0.08 (0.08)	0.326	0.10 (0.08)	0.204	−0.07 (0.08)	0.379	−0.04 (0.08)	0.592	−0.09 (0.08)	0.236	−0.06 (0.08)	0.431
Area deprivation	Higher	0.00 (0.02)	0.993	0.01 (0.02)	0.733	−0.01 (0.02)	0.466	−0.01 (0.02)	0.475	−0.02 (0.02)	0.413	−0.02 (0.02)	0.437
Remoteness (large urban)	Other urban	0.07 (0.07)	0.322	0.07 (0.07)	0.305	0.06 (0.06)	0.350	0.05 (0.06)	0.427	0.00 (0.06)	0.992	0.00 (0.06)	0.962
	Accessible	−0.07 (0.08)	0.354	−0.09 (0.08)	0.235	−0.03 (0.05)	0.561	−0.04 (0.05)	0.426	−0.04 (0.07)	0.509	−0.06 (0.06)	0.371
	Remote	−**0.20 (0.09)**	**0.027**	−**0.22 (0.09)**	**0.013**	−**0.23 (0.07)**	**0.001**	**−0.24 (0.08)**	**0.001**	−**0.27 (0.06)**	**<0.001**	−**0.27 (0.06)**	**<0.001**
Parenting (46–70 months)													
Home learning	More frequent			−0.04 (0.04)	0.272			**0.11 (0.03)**	**<0.001**			**0.08 (0.03)**	**0.005**
Dysfunctional parenting	Higher			−**0.10 (0.04)**	**0.012**			−**0.11 (0.04)**	**0.002**			−**0.16 (0.04)**	**<0.001**
Protectiveness	Higher			−0.07 (0.04)	0.059			0.01 (0.03)	0.774			**0.08 (0.03)**	**0.012**

Measures were mutually adjusted, and also adjusted for child gender, birth order, health (10–22 months), developmental delay (22 months) and cognitive score (34 months); mother’s ethnicity, age at birth of child and low physical health (10 months); and number of children in the household (10 months). Figures in bold show associations that were statistically significant at the *p* < 0.05 level

**Fig. 1:**
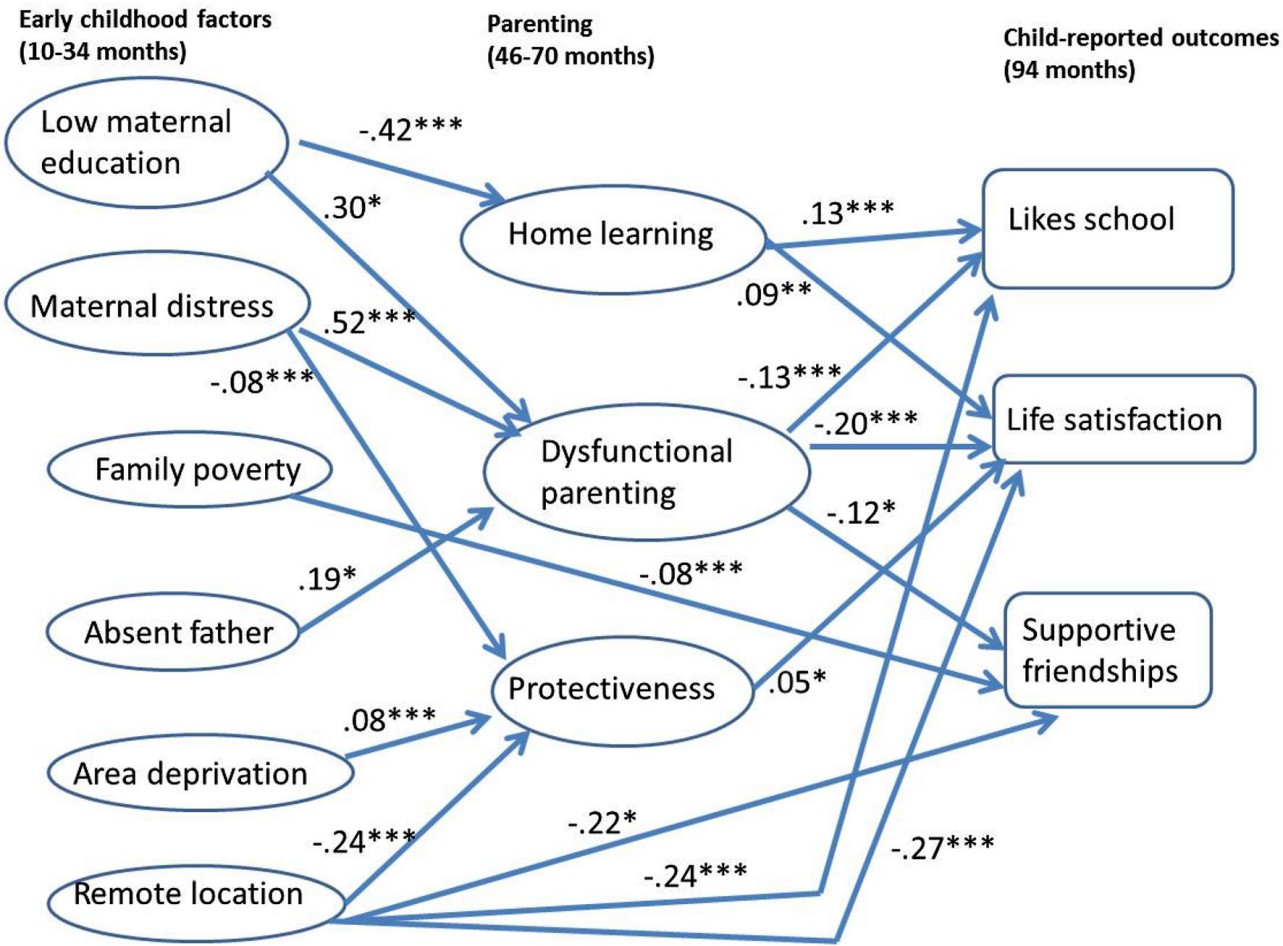
Path model of associations between early childhood factors, parenting and children’s subjective well-being. Model as for Table 3. This figure omits non-significant associations between measures shown here, and all associations between measures shown and additional covariates: child gender, birth order, health (10–22 months), developmental delay (22 months) and cognitive score (34 months); mother’s ethnicity, age at birth of child and low physical health (10 months); and number of children in the household. With the exception of pathways from binary measures (low maternal education, father absence, remoteness), to allow comparison of pathways figures shown here (unlike Table 3) represent standardised coefficients with *p < 0.05, **p < 0.01, ***p < 0.001

In stage 2, path models allowed for associations between early childhood factors and child outcomes via parenting, as well as direct links between early childhood factors and outcomes. Figure 1 shows significant pathways in the final model: for ease of comparison, standardised coefficients are provided (except for paths from binary measures). Unstandardised coefficients for direct effects of early childhood factors and parenting are shown in Table 3, stage 2. Home learning was predictive of liking school and greater life satisfaction; dysfunctional parenting had significant negative associations with all three outcomes; while protectiveness predicted greater life satisfaction. After allowing for parenting, direct effects of maternal distress were attenuated to non-significance, but the effects of poverty and remote location observed in stage 1 were largely unchanged.

Despite the non-significant direct effect of maternal distress in the final model, it had indirect associations with children’s more negative evaluations of friends, school and life, via more dysfunctional parenting (Fig. 1; Table 4). Table 4 also shows smaller indirect effects of low maternal education and father absence on lower life satisfaction via dysfunctional parenting. There were additional negative indirect effects on well-being from low maternal education via lower home learning. Lastly, opposing pathways via protectiveness were found. While area deprivation produced a small positive indirect effect on life satisfaction via greater protectiveness, maternal distress and remote location both produced corresponding negative indirect effects. A sensitivity analysis (not shown) exploring the effect of restricting indicators of the maternal distress construct to measures of low mental health (i.e. excluding the two indicators of illegal drug use) found that results were unchanged.

**Table 4:** Indirect effects (via parenting) of early childhood factors on 7-year-olds’ well-being

Early childhood	Pathway	Child-reported outcomes at 94 months					
		Supportive friends		Liking school		Life satisfaction	
		Estimate (SE)	*p*	Estimate (SE)	*p*	Estimate (SE)	*p*
Low maternal education	Via home learning	0.015 (0.016)	0.335	**−0.044 (0.015)**	**0.004**	**−0.034 (0.013)**	**0.007**
	Via dysfunctional parenting	−0.029 (0.016)	0.078	−0.032 (0.017)	0.059	**−0.049 (0.025)**	**0.049**
Maternal distress	Via protectiveness	0.004 (0.003)	0.139	0.000 (0.002)	0.762	**−0.005 (0.002)**	**0.024**
	Via dysfunctional parenting	**−0.063 (0.027)**	**0.019**	**−0.071 (0.023)**	**0.003**	**−0.106 (0.026)**	**<0.001**
Absent father	Via dysfunctional parenting	−0.018 (0.012)	0.131	−0.020 (0.012)	0.079	**−0.031 (0.016**)	**0.049**
Area deprivation	Via protectiveness	−0.003 (0.002)	0.144	0.000 (0.001)	0.765	**0.003 (0.002)**	**0.041**
Remote location	Via protectiveness	0.016 (0.010)	0.085	−0.002 (0.007)	0.763	**−0.020 (0.009)**	**0.022**

Unstandardised coefficients are shown. Table omits indirect effects not statistically significant at *p* < 0.05 for all child outcomes (this includes all indirect effects from family poverty). Model adjusted for child gender, birth order, health (10–22 months), developmental delay (22 months) and cognitive score (34 months); mother’s ethnicity, age at birth of child and low physical health (10 months), and number of children in the household

Figures in bold show indirect paths that were statistically significant at the *p* < 0.05 level

A supplementary analysis suggested broad agreement between mother-reported child adjustment at 7 years and child subjective well-being constructs although mother-reported peer and emotional problems did not map neatly on to (respectively) child-reported supportive friendships and life satisfaction. Mother-reported school adjustment was more strongly associated with the child’s own reports of liking school (0.24), than with the other two child constructs (−0.11, −0.16). Mother-reported peer and emotional problems were both negatively associated with child-reported supportive friendships (−0.42, −0.25, respectively) and life satisfaction (−0.31, −0.24, respectively), and less strongly with liking school (−0.12, −0.09). Multivariable analysis (supplementary file S2, stage 1) showed independent effects of all main predictors on one or more mother-reported outcomes, with larger effect sizes than for child-reported outcomes. However, there was no effect of remoteness on mother-reported school adjustment. In stage 2 models, effects of dysfunctional parenting and home learning were similar to those seen for child-reported outcomes, but protectiveness had no effect. Effects of maternal distress were attenuated with the inclusion of dysfunctional parenting, as for child-reported outcomes. There were more remaining significant effects of other early childhood factors. These included effects of low maternal education, family poverty, area deprivation and remoteness on increased peer problems; absent father on reduced school adjustment; and lower maternal education and absent father on increased emotional problems.

## Discussion

This study presents unique longitudinal data on the impact of early childhood disadvantage and parenting on 7-year-olds’ own evaluations of friendships, school and life satisfaction. In terms of early childhood influences, our findings suggested that early maternal distress, family poverty and geographical location were predictive of children’s later subjective well-being although the effects of maternal distress were conveyed indirectly, largely via more dysfunctional parenting. The harmful role of dysfunctional parenting emerged most strongly, across children’s ability to form friendships, enjoyment of school and life satisfaction. Home learning predicted greater school enjoyment, and was also important (along with protectiveness) for overall life satisfaction.

Our findings for child-reported outcomes were supported by findings for comparable mother-reported outcomes, particularly with regard to the role of maternal distress, poverty, dysfunctional parenting and home learning. Differences in the strength of associations found are likely to reflect the effects of shared method variance, with mothers reporting outcomes as well as predictors, as well as the imperfect correspondence between attributes assessed by mother and child at the 7-year-old interview. Nonetheless, discrepancies in the pattern seen for child- and mother-reported outcomes with respect to the effects of remoteness and protectiveness support the idea that children’s perspectives will not necessarily tally with those of their parents.

Our study points to the importance of parenting processes for young children’s subjective well-being, adding strength to limited evidence from studies of adolescent life satisfaction [16, 60, 61]. It also indicates the role of parenting in mediating more distal (earlier) influences on the child. The parenting pathways found for dysfunctional parenting corroborate existing research on mechanisms linking family-level risk with child socio-emotional problems via parent–child conflict, home disorganisation and parenting stress [11, 40–43]. Our finding of a path from maternal education to school adjustment and life satisfaction via home learning supports the idea that parental provision of stimulating experiences and materials is linked with socio-emotional adjustment [36–39], as well as with future academic achievement although such activities could also reflect other differences in parenting style and expectations. To our knowledge, parental protectiveness has not been previously linked with child subjective well-being. Its effects on life satisfaction may relate to other aspects of family processes important for socio-emotional adjustment, including attachment [62] and neglectful parenting [63]. However, the small indirect paths found from neighbourhood deprivation via greater protectiveness, and from remoteness via lower protectiveness, are likely to reflect area variation in parental perception of risk [45, 46] and strategies for monitoring children’s outdoor play, as found in some research linking neighbourhood danger to greater parental monitoring of teenagers [64].

Most of the effects of remote location on all three subjective well-being measures could not be accounted for by parenting, suggesting that further explanation is required. The remote study population was highly stable (88 % remaining in remote areas at 7 years), and since previous research suggests older children are more sensitive to the limitations of rural neighbourhoods, it is likely that associations reflect the influence of current location. Others have found that parental perceptions of greater safety and opportunity for outdoor play in rural areas often contrast with children’s own negative views of the countryside [52]. However, we found an effect of remote location (including small towns far from major urban centres) rather than rurality. Contributing factors may include fewer friendship choices, increased travel times and media influences exposing children to wider horizons: more research is required to establish these, and whether our finding foreshadows effects of rurality on lower mental health found for teenagers and young adults [32, 65].

Parenting also failed to account for the effect of poverty. In our study, negative effects of poverty were confined to children’s perceived friendship quality, in contrast to more pervasive effects on older children’s subjective well-being [19]. Our finding may reflect the effect of persistent poverty on 7-year-olds, as two-thirds of families reporting low income in the early years were in similar circumstances at 7 years. Effects may relate to even young children’s perceptions of low social status through inability to afford leisure activities or the latest clothes and gadgets [49].

Limitations of this study include reliance on mothers for sensitive information subject to social desirability and other biases, the sample’s low ethnic diversity, and a lack of information on fathers and on parenting during infancy and toddlerhood. Strengths include the large sample, representative of the Scottish population at baseline, and the use of survey weights to counteract the effect of differential attrition of more disadvantaged groups over the course of the study.

Further data are required from young children to assess other important aspects of subjective well-being, particularly regarding family relationships, and to pursue the impact of both early childhood and concurrent family disadvantage on older age groups. Our study points to the need for a more rounded picture of children’s socio-emotional development, to allow for some differences between child and adult perspectives. It underlines the continued need, in addition to UK social policies reducing child poverty from the late 1990s, to target resources for mothers with poor mental health. It also underscores the damaging effects, from the child’s as well as the parent’s perspective, of dysfunctional parenting. This echoes other findings of spillover effects of family stress and conflict on multiple aspects of children’s lives [66], and suggests that helping parents to develop better skills to support their child at home and school would improve children’s feelings of well-being.

## Electronic supplementary material

Below is the link to the electronic supplementary material.

Supplementary material 1 (DOCX 26 kb)

Supplementary material 2 (DOCX 36 kb)
