# Supplementary material for: What shapes 7-year-olds’ subjective well-being? Prospective analysis of early childhood and parenting using the Growing Up in Scotland study
Source: Soc Psychiatry Psychiatr Epidemiol. 2016 Jun 30;51(10):1417–28. doi: 10.1007/s00127-016-1246-z (PMC5047922; doi:10.1007/s00127-016-1246-z)
Supplement: Supplementary file 1 — Supplementary material 1 (DOCX 26 kb) [file 127_2016_1246_MOESM1_ESM.docx]

**Supplementary file 2**

Article **What shapes seven-year-olds’ subjective well-being? Prospective analysis of early childhood and parenting using the Growing Up in Scotland Study**

**Alison Parkes^a^, Helen Sweeting^a^, and Daniel Wight^a^**

**For Social Psychiatry and Psychiatric Epidemiology**

^a^MRC/ CSO Social and Public Health Sciences Unit, University of Glasgow, United Kingdom.

Corresponding author: Alison Parkes, Email:Alison.parkes@glasgow.ac.uk.

**Early childhood and parenting measures: adjusted associations with mother-reported seven-year olds' socio-emotional adjustment: results of adjusted models**

|  |  | **Mother-reported outcomes at 94 months** | | | | | | | | | | | |
| --- | --- | --- | --- | --- | --- | --- | --- | --- | --- | --- | --- | --- | --- |
|  |  | **Peer relationship problems** | | | | **School adjustment** | | | | **Emotional problems** | | | |
| **Measure (with reference group for categorical measures)** | **Effect/contrast** | **Stage 1** | | **Stage 2** | | **Stage 1** | | **Stage 2** | | **Stage 1** | | **Stage 2** | |
|  |  | **β (SE)** | ***p*** | **β (SE)** | ***p*** | **β (SE)** | ***p*** | **β (SE)** | ***p*** | **β (SE)** | ***p*** | **β (SE)** | ***p*** |
| **Early childhood (10-34 months)** |  |  |  |  |  |  |  |  |  |  |  |  |  |
| Maternal education (degree) | advanced | 0.07 (0.11) | 0.485 | 0.13 (0.10) | 0.177 | 0.00 (0.05) | 0.992 | -0.01 (0.05) | 0.843 | 0.09 (0.10) | 0.330 | 0.15 (0.09) | 0.096 |
|  | intermediate | **0.28 (0.12)** | **0.019** | **0.30 (0.12)** | **0.009** | -0.09 (0.05) | 0.117 | -0.06 (0.05) | 0.275 | 0.24 (0.12) | 0.037 | **0.23 (0.11)** | **0.042** |
|  | low | **0.48 (0.15)** | **0.001** | **0.37 (0.13)** | **0.006** | -0.09 (0.06) | 0.183 | 0.01 (0.06) | 0.929 | **0.51 (0.12)** | **<0.001** | **0.32 (0.11)** | **0.005** |
|  |  |  |  |  |  |  |  |  |  |  |  |  |  |
| Maternal distress | higher | **0.26 (0.04)** | **<0.001** | 0.07 (0.07) | 0.279 | **-0.08 (0.03)** | **0.003** | 0.01 (0.04) | 0.809 | **0.34 (0.06)** | **<0.001** | 0.08 (0.08) | 0.360 |
|  |  |  |  |  |  |  |  |  |  |  |  |  |  |
| Family poverty | higher | **0.16 (0.05)** | **0.001** | **0.13 (0.05)** | **0.008** | **-0.05 (0.02)** | **0.020** | -0.04 (0.02) | 0.057 | **0.15 (0.06)** | **0.015** | 0.11 (0.06) | 0.058 |
|  |  |  |  |  |  |  |  |  |  |  |  |  |  |
| Absent father (no) | yes | **0.25 (0.11)** | **0.019** | 0.18 (0.10) | 0.060 | **-0.15 (0.06)** | **0.005** | **-0.12 (0.05)** | **0.026** | **0.29 (0.10)** | **0.004** | **0.20 (0.09)** | **0.032** |
|  |  |  |  |  |  |  |  |  |  |  |  |  |  |
| Area deprivation | higher | **0.08 (0.03)** | **0.007** | **0.07 (0.03)** | **0.028** | -0.02 (0.02) | 0.158 | -0.02 (0.02) | 0.260 | 0.02 (0.03) | 0.489 | 0.01 (0.03) | 0.772 |
|  |  |  |  |  |  |  |  |  |  |  |  |  |  |
| Remoteness (large urban) | other urban | -0.03 (0.12) | 0.768 | -0.02 (0.12) | 0.859 | **0.16 (0.06)** | **0.005** | **0.14 (0.06)** | **0.012** | -0.06 (0.09) | 0.537 | -0.03 (0.09) | 0.782 |
|  | accessible | -0.06 (0.10) | 0.574 | -0.01 (0.09) | 0.933 | 0.07 (0.05) | 0.203 | 0.05 (0.05) | 0.346 | -0.04 (0.11) | 0.692 | 0.01 (0.10) | 0.894 |
|  | remote | **0.31 (0.09)** | **0.001** | **0.33 (0.10)** | **0.001** | 0.08 (0.09) | 0.376 | 0.07 (0.08) | 0.424 | 0.23 (0.13) | 0.073 | 0.24 (0.13) | 0.075 |
|  |  |  |  |  |  |  |  |  |  |  |  |  |  |
| **Parenting (46-70 months)** |  |  |  |  |  |  |  |  |  |  |  |  |  |
|  |  |  |  |  |  |  |  |  |  |  |  |  |  |
| Home learning activities | more frequent |  |  | 0.04 (0.05) | 0.338 |  |  | **0.10 (0.02)** | **<0.001** |  |  | -0.08 (0.05) | 0.080 |
|  |  |  |  |  |  |  |  |  |  |  |  |  |  |
| Dysfunctional parenting (lower) | higher |  |  | **0.31 (0.04)** | **<0.001** |  |  | **-0.12 (0.02)** | **<0.001** |  |  | **0.40 (0.05)** | **<0.001** |
|  |  |  |  |  |  |  |  |  |  |  |  |  |  |
| Protectiveness | higher |  |  | 0.04 (0.04) | 0.319 |  |  | -0.03 (0.03) | 0.306 |  |  | 0.00 (0.05) | 0.955 |
|  |  |  |  |  |  |  |  |  |  |  |  |  |  |

Note: Measures were mutually adjusted, and also adjusted for child gender, birth order, health (10-22 months), developmental delay (22 months) and cognitive score (34 months); mother's ethnicity, age at birth of child and low physical health (10 months); and number of children in the household. Figures in bold show associations that were statistically significant at the p<0.05 level.
