# Supplementary material for: What shapes 7-year-olds’ subjective well-being? Prospective analysis of early childhood and parenting using the Growing Up in Scotland study
Source: Soc Psychiatry Psychiatr Epidemiol. 2016 Jun 30;51(10):1417–28. doi: 10.1007/s00127-016-1246-z (PMC5047922; doi:10.1007/s00127-016-1246-z)
Supplement: Supplementary file 2 — Supplementary material 2 (DOCX 36 kb) [file 127_2016_1246_MOESM2_ESM.docx]

Article: **What shapes seven-year-olds’ subjective well-being? Prospective analysis of early childhood and parenting using the Growing Up in Scotland Study.**

**For Social Psychiatry and Psychiatric Epidemiology**

**Alison Parkes^a^, Helen Sweeting^a^, and Daniel Wight^a^**

^a^MRC/ CSO Social and Public Health Sciences Unit, University of Glasgow, United Kingdom.

Corresponding author: Alison Parkes, Email:Alison.parkes@glasgow.ac.uk.

**Details of measures and sample information (note, all information provided by mothers, with the exception of child cognitive score)**

| **Construct** | **Indicator measures**  **(for latent constructs)** | **Time period (s) when measured** | **Details** | **Mean (SE) or %** | **Factor loading(s) for latent construct (where applicable)** |
| --- | --- | --- | --- | --- | --- |
| **Child gender** |  | 10 months | Coded as 0=male, 1=female. | 49.4% female | - |
| **Birth order** |  | 10 months | Recoded as 0=not first born, 1=first born | 48.2% first born | - |
| **Child general health** |  | 10-22 months | Reported using a 5-point scale (1=very good to 5=very bad), with the average score used. | 1.33 (0.01) |  |
| **Child developmental delay** |  | 22 months | Assessed using the parent-reported Communication and Symbolic Behavior Scales Developmental Profile [1], using the recommended cut-off. | 6.1% |  |
| **Child cognitive score** |  | 38 months | Non-verbal reasoning was assessed by trained researchers using the Picture Similarities subscale of the British Ability Scales (BAS) II [2] Scores were standardised according to age-related norms | 51.06 (0.19) |  |
| **Mother’s age at birth of child** |  | 10 months | Mothers were grouped into four age bands: under 20, 20-29, 30-39 and 40 plus years. | Under 20- 3.2%, 20-29 yrs – 35.3%, 30-39 yrs, 57.8%, 40+ - 3.7% |  |
| **Maternal educational level** |  | 10 months | Using the Scottish Credit and Qualifications framework, mothers’ qualifications were divided into four groups: degree-level, advanced (Scottish Highers or equivalent), intermediate (upper-level Scottish Standard grades, or equivalent) or low (lower-level Scottish Standard grades or equivalent, or no qualifications). For more information, see <http://www.sqa.org.uk/sqa/4596.557.html> | Degree- 32.6%, advanced – 34.5%, intermediate -23.1%, low – 9.8% |  |
| **Maternal ethnic group** |  | 10 months | Coded according to Scottish National Census classification. Recoded as 0=white, 1=Ethnic Minority. | 2.4% | - |
| **Maternal distress** | Low maternal mental health | 10,22,34 months | At 10 and 34 months, this was based on the Mental MCS-12 Scale [3], where the threshold for low health was a score ≤1 standard deviation below the mean. At 22 months, this was based on the combined Depression and Stress subscales of the DASS scale (REF), where the threshold for low health was a score ≥ 1 standard deviation above the mean. Coded 0=no, 1=yes. | 13.6%, 15.2%, 14.8% | 0.71, 0.78, 0.76 |
|  | Drug use | 10, 34 months | Illegal drug use in the past 12 month, based on 9 items about any use of cannabis, amphetamines, cocaine, crack, ecstasy, heroin, methadone, LSD, another illegal drug. Coded 0=no, 1=yes. | 3.1%, 3.3% | 0.76, 0.69 |
| **Mother’s low physical health** |  | 10 months | Physical health was measured using the Short Form Health Survey (SF-12) physical health subscale [4], and low health was defined as mothers with a score <1 SD from the mean. | 10.9% |  |
| **Family poverty** | Low family income | 10,22,34 months | Family income was based on an item about the total household income from all sources  before tax. Responses were on a 17-point banded scale. Income was equivalised to take account of household size and composition. Low income was defined as ≤60% of UK median income, and coded as 0=no, 1=yes. | 21.8%, 19.4%, 19.3% | 0.93, 0.89, 0.93 |
|  | Workless household | 10,22,34 months | Mother and (where applicable) resident partner not in employment. Coded as 0=no, 1=yes. | 9.1%, 8.9%, 8.6% | 0.95, 0.99, 0.96 |
| **Father absence** |  | 10,22,34 months | Father not resident in household at one or more of 10,22 and 34 months surveys | 16.1% |  |
| **Family size** |  | 10 months | Number of children (under 16 years old) in household. Families with four or more children were combined. | One-47.4%, two-35.8%, three-13.4%, four or more -3.4% | - |
| **Area deprivation** |  | 10 months | Household postcode was linked to quintiles (q) of the Scottish Index of Multiple Deprivation 2006 [5] (here ranked from 1, least deprived to 5, most deprived). This classifies small areas on the basis of householders’ income, employment, health, education, geographic access to services and housing. | q1- 22.8%, q2-22.2%, q3-22.5%, q4-16.7%, q5-15.8% |  |
| **Rurality and remoteness** |  | 10 months | Household postcode location used the six-part Scottish urban-rural indicator (see http://www.scotland.gov.uk/Topics/Statistics/About/Methodology/UrbanRuralClassification) . This divides areas into large urban areas (settlements of 125,000 people), other urban areas (settlements 10,000-<125,000), accessible small towns (settlements of 3,000-<10,000), remote small towns, accessible rural and remote rural. Accessible areas are those within a 30 minute drive time of a settlement of 10,000 or more, while for remote areas the drive time is more than 30 minutes. Two groupings of non-urban areas were explored. The first combined small towns in accessible and remote areas, and combined accessible and remote rural locations, to produce a small town vs rural grouping. The second combined accessible small towns and rural areas, and combined remote small towns and rural areas to give an accessible vs remote grouping. | At 10 months: Large urban 36%, other urban 29%, small town 13%, rural 22%, accessible 26%, remote 9% | - |
| **Dysfunctional parenting** | Parent-child conflict | 58 months | A standardised score using 7 items from Pianta scale [6], Cronbach alpha=0.82. Conflict was measured using seven items, for example: “(Child’s name) and I always seem to be struggling with each other” using a 5-point scale (1=definitely does not apply to 5=definitely applies). | 0.00 (0.01) | 0.55 |
|  | Household organisation | 58 months | A standardised score using 3 items from the confusion, hubbub, and order scale ([7]), Cronbach alpha=0.65. Agreement with : “It’s really disorganised in our home”, “You can’t hear yourself think in our home” and “The atmosphere in our home is calm” (item reversed), using a 5-point scale (1=strongly disagree, to 5= strongly agree). | 0.00 (0.01) | 0.59 |
|  | Parenting stress | 58 months | A standardised score using four items from the Parental Stress scale ([8]), Cronbach alpha 0.71. Agreement with: "“Being a parent is harder than I thought it would be”, "”I feel trapped by my responsibilities as a parent”, “I find that taking care of my child(ren) is much more work than pleasure”, "“I often feel tired, worn out, or exhausted from raising a family” using a 5-point scale (1=strongly disagree, to 5=strongly agree). | 0.00 (0.01) | 0.66 |
| **Protectiveness** |  | 46 months | A standardised scale based on four items from the Parent Supervision Attributes Profile Questionnaire [9] Cronbach alpha=0.67. Agreement with items asked in relation to looking after the child playing outside: "I stay close enough to my child so that I can get to him/her quickly", “I think of all the dangerous things that could happen”, “I make sure I know where my child is and what he/she is doing”, “I feel very protective of my child” using a 5-point scale (1=strongly disagree, to 5=strongly agree). | 0.00 (0.02) | - |
| **Home learning** |  | 46, 58 and 70 months | Frequency of four home learning activities used standardised scales based on information at three timepoints: looking at books or read stories (Cronbach alpha 0.65), activities involving painting or drawing (Cronbach alpha=0.61); reciting nursery rhymes or sung songs (Cronbach alpha=0.64); playing at recognising letters, words, numbers or shapes (Cronbach alpha=0.70), scale based on the number of days in the past week (range 0-7). | 0.00 (0.02), 0.00 (0.02), 0.00 (0.02), 0.00 (0.02) | 0.59, 0.63, 0.48, 0.60 |

**References**

1. Wetherby, AM, Allen, L, Cleary, J, Kublin, K, and Goldstein, H. Validity and reliability of the communication and symbolic behavior scales developmental profile with very young children*.* Journal of Speech, Language and Hearing Research 2002; **45**(6): 1202-18.doi:

2. Elliott, CD, Smith, P, and McCulloch, K, British Ability Scales second edition (BAS II): Administration and scoring manual. 1996 London: NFER-Nelson.

3. Ware, JE, Jr., Kosinski, M, and Keller, SD. A 12-Item Short-Form Health Survey: Construction of Scales and Preliminary Tests of Reliability and Validity*.* Medical Care 1996; **34**(3): 220-233.doi:

4. Jenkinson, C and Layte, R. Development and testing of the UK SF-12 (short form health survey)*.* Journal of Health Services Research & Policy 1997; **2**(1): 14-8.doi:

5. Scottish Executive, Scottish Index of Multiple Deprivation, in *Scottish Executive National Statistics Publication*. 2006, Scottish Executive: Edinburgh.

6. Pianta, RC, Child-Parent Relationship Scale. 1992 Charlottesville, VA: Unpublished measure, University of Virginia.

7. Matheny, JAP, Wachs, TD, Ludwig, JL, and Phillips, K. Bringing order out of chaos: Psychometric characteristics of the confusion, hubbub, and order scale*.* Journal of Applied Developmental Psychology 1995; **16**(3): 429-444.doi:

8. Berry, JO and Jones, WH. The Parental Stress Scale: Initial Psychometric Evidence*.* Journal of Social and Personal Relationships 1995; **12**(3): 463-472.doi:10.1177/0265407595123009

9. Morrongiello, BA and Corbett, M. The Parent Supervision Attributes Profile Questionnaire: a measure of supervision relevant to children’s risk of unintentional injury*.* Injury Prevention 2006; **12**(1): 19-23.doi:10.1136/ip.2005.008862
